# Local Effects of Chloroform and Other Anæsthetic Agents

**Published:** 1848-08

**Authors:** 


### YORKSHIRE BRANCH OF THE PROVINCIAL MEDICAL AND SURGICAL ASSOCIATION.

*Local Effects of Chloroform and other Anæsthetic Agents.*—Mr. Nunnelly stated that for many months he had been engaged in making experimental researches on these agents, with a view to ascertain, as far as possible, the modus operandi, the doses which may be borne with impunity, and the different modes of application; as well as in case of an overdose, the best means to be adopted to counteract it. His experiments have not merely extended to the common anæsthetic agents employed, such as ether and chloroform, but he has been endeavoring to ascertain whether or not there may be some others, which may either be more safely demonstrated, or may possess still greater advantages than the usual agent employed ; which appeared to him to be by no means impossible, inasmuch as, so far as he could ascertain, the selection of those now employed, rather than others, seemed to have depended rather upon accident than deduction from experimental research, proving them to be in all respects the best. He stated that he believed it not improbable that it would ultimately be found, that all those preparations which have a *radical basis*, (in the language of modern chemistry,) such as acetic ether, bisulphuret of carbon, aldehyde, and many others of an analogous character, upon some of which he had made extensive experiments, would possess similar properties on the animal economy.

Mr. Nunnelly was also prepared to state, *that chloroform appeared to be the most deleterious to life*, to require the greatest care in the administration, and that the boundary up to a fatal dose is by no means well marked—that of two animals in apparently the same condition, the same dose being given in precisely the same way to both, the one will speedily die, while the other will bear it with impunity,—that from the effects observed, he has reason to think the ultimate effects must be dissimilar to those produced by prussic acid,—that to some animals, as for instance the newt, the frog, the toad, some fish, slugs, snails, and some insects, the effects are *more* rapidly fatal than prussic acid of Scheele's strength ; and that even in higher animals, when under the influence of an incomplete dose, or recovering from the effects of a large dose of ether, chloroform, or prussic acid, the phenomena are in many respects very similar,—and further, that the numerous *post-mortem* examinations which he has made, fully corroborated this opinion. He stated that acetic ether, with which he had made numerous experiments, possesses very considerable anæsthetic powers,—the bisulphuret of carbon does also possess to some extent similar power, and so far as his experiments go, it is very important to add, that this power is of a safe character, the animal speedily recovering. But of all these remedies he believes that sulphuric ether will be found to be the safest and least noxious to life.

On these points Mr. Nunnelly intends hereafter to lay his experiments, already very numerous and varied, before the profession. His chief object on the present occasion was to call the attention of the profession to experiments proving, as he thinks, the value and safety of a *new mode* of administering these agents, and to show that the action of all, or most of these agents might be produced *locally by local application*, the censorium being unaffected, consciousness being retained, and the limbs not subjected to their influence being unaffected. He stated that either by *immersion* in a small quantity, or by the *vapour applied* merely for a limited period, a limb may be rendered *perfectly motionless and senseless*, and what may be an additional advantage, *fixed in any desired position.* He had immersed his finger in these fluids for about half an hour, or an hour, and at the end of this period the finger was nearly powerless and insensible, and that it was forty-eight hours before the effects entirely disappeared, a sensation of heat and discomfort extending along the tract of the nerves to the axilla. Before operating on a difficult case for artificial pupil, he had applied for twenty minutes a small quantity of the vapour of chloroform to the eye by means of a small jar which accurately fitted the orbit, with the effect of rendering the parts nearly insensible. The first effect of these agents when locally applied is to produce redness, heat, and smarting, which subside, followed by swelling and redness of the integuments, which remain for some time. Mr. Nunnelly stated that he could completely paralyse any limb of frogs or toads by immersion or exposure to the vapour, in about five minutes or less ; and he mentioned, as a curious fact, that if the exposure to the influence were continued longer than was sufficient to produce a local effect, this influence extended to the corresponding limb of the other side : thus, for instance, if one hind leg became *too much* influenced, the other hind leg partook of the same effect—if the fore leg were too much affected, then the other fore leg became so likewise, and subsequently, the whole body—a result which Mr. Nunnelly mentioned as strongly corroborative of his experiments with prussic acid, as detailed in the last volume of the "Provincial Transactions," and strongly supporting the opinions of Dr. Marshall Hall on "reflex action." By this new mode of application to the hind legs of rabbits he had also been able to amputate the toes without the least indication of feeling.

These views were illustrated by a series of experiments on frogs and toads, in which, after immersion for a few minutes, the limbs became insensible, and were amputated in repeated portions without any symptoms of pain whatever.

[We are indebted to Mr. Nunnelly for the foregoing account, (taken from the forthcoming volume of Mr. Braithwaite's "Retrospect,") and also for a mode of performing the experiments on animals, from which the following is an extract :]—

"For showing the local effect, nothing is better adapted than the living leg or legs of a frog. All that is necessary is to put a few drops of the agent we wish to ascertain the effect of, into an ounce, ounce and a half, or two ounce vial, according to the size of the frog, having such a mouth as to receive the limb, and just fit around the upper part of the thigh, without, however, compressing it, and in order to avoid the vapour escaping, then passing round a few folds of a strip of linen or other bandage. If it is wished to see the effects of the vapour, the bottle should be sufficiently long not to allow the toes to touch the fluid, and the bottle should be kept quite still in an upright position ; but if it be wished to apply the fluid itself, then the toes should touch the bottom, or the bottle be placed from time to time on the side in order to allow the fluid to pass over the limb, by which proceeding but little of the substance is required. At first the creature struggles, the fluid evidently acting as a stimulant, and at that time a little effort is required to keep it in position ; presently it will remain *in situ*, for the reversed member becomes still and motionless. In from two to five minutes the full effect is produced, and the limb is perfectly insensible. If one leg be kept in too long, the other hind leg is affected, or the whole body is brought under the influence of it; when the experiment is less satisfactory, and the animal is liable to die. After the application the limb remains for some time swollen, and the colour of the skin is altered.

"In the case of other animals, the time necessary for immersion, before complete insensibility takes place, will vary with circumstances as to the temperature, but more especially the aptitude of the integument for absorption, and the nature of its covering. Thus, if the skin be hard, the epidermis thick, with the hair stiff and long, then a considerable period will be required. In the case of a rabbit, the other night, after an hour's immersion, I cut off the whole of the toes in succession, and then amputated the entire foot, cutting through the metatarsal, and then the tarsal bones, the creature lying upon my knees in the most quiescent state possible, there not being the least disturbance of the countenance during these operations. But the moment either of the other limbs were touched, and also what is of great interest, the *same limb above where the chloroform had reached*, it at once started up with every indication of pain, shewing how precisely the effect may be limited. The same operations I have also performed upon a kitten, and upon two dogs, (old;) these were, as might be expected after a similar length of immersion, though considerable diminution of sensibility was produced, not rendered entirely devoid of feeling.

"I may mention that to-day, (June 22nd,) I have operated upon a staphylatomatous eye, in a man from a distance, after the local action of chloroform, when I removed the entire cornea with a small portion of sclerotic coat and iris, with hardly any infliction of pain; and what is singular, so completely were the muscles of the globe affected, that for some time afterwards the remains of the eye did not collapse, although their attachments were not cut through. The fluid had been applied longer than necessary, or I had intended, as after having given directions for it to be applied until my arrival, I was detained away longer than I had expected.

"I have been making experiments upon two new agents, both of which I find to possess considerable anæsthetic property, and one of which I believe will prove to be superior in every respect to chloroform."—*Provincial Medical and Surgical Journal.*

